# Psychometric Properties of the Brazilian Version of the Quality of Dying and Death for Adult Family Members of ICU Patients

**DOI:** 10.3390/ijerph20065034

**Published:** 2023-03-13

**Authors:** Cariston Rodrigo Benichel, Silmara Meneguin, Camila Fernandes Pollo, Mariele Gobo Oliveira, Cesar de Oliveira

**Affiliations:** 1 Department of Nursing, Botucatu Medical School, Paulista State University, São Paulo 18618687, Brazil; 2 Department of Epidemiology & Public Health, University College London, London WC1E 6BT, UK; c.oliveira@ucl.ac.uk

**Keywords:** validation studies, psychometrics, death, intensive care units

## Abstract

Death is a complex, subjective phenomenon that requires an understanding of experiences to be qualified to provide care during the end-of-life process. This study aimed to analyze the psychometric properties of the Portuguese version (Brazil) of the Quality of Dying and Death (QODD) scale on family members of patients who died in adult intensive care units. A methodological study was conducted with 326 family members of patients that died in three ICUs of public hospitals in the state of São Paulo, Brazil. For this study, the QODD 3.2a (25 items and six domains) was administered during the period from December 2020 to March 2022. The analysis was performed using the classic theory of the tests and the goodness of fit of the model was tested using confirmatory factor analysis. We have used Spearman’s correlation coefficients between the scores of the overall scale and domains. Cronbach’s alpha coefficient and the intraclass correlation coefficient (ICC) were used for the evaluation of internal consistency and temporal stability, respectively. The Horn’s parallel analysis indicated two factors that were not confirmed in the exploratory factor analysis. A single factor retained 18 of the initial 25 items and the analysis of the goodness of fit to the unidimensional model resulted in the following: CFI = 0.7545, TLI = 0.690, chi-squared = 767.33, df = 135, RMSEA = 0.121 with 90%CI, and *p* = 5.04409. The inter-item correlations indicated a predominance of weak correlations among the items of the instrument. The items with the largest number of moderate correlations were questions 13b, 9b, and 10b and a strong correlation was found between questions 15b and 16b. Cronbach’s alpha coefficient was 0.8 and the ICC was 0.9. The Quality of Dying and Death—Version 3.2a (intensive therapy) in Brazilian Portuguese has a unidimensional structure and acceptable reliability. However, it did not obtain a good fit to the proposed factorial model.

## 1. Introduction

The process of dying has always been permeated with numerous reflections involving subjectivity, the concept of a good death, and end-of-life care. Aspects such as the transference of the patient to the hospital setting, the anxiety of those involved face the care provided by the health team bring new meanings that permeates human life [1,2]. 

In this context, the literature shows that hospitalization in intensive care is often characterized by end-of-life experiences influenced by social, cultural, and religious beliefs and involves a loss of routine and autonomy and the occurrence of conflict and painful episodes. These include interventional procedures such as intubation and mechanic ventilation, dialysis support, and cardiorespiratory resuscitation maneuver, as well as difficult symptoms such as pain, difficulty breathing, disorientation, and extreme conditions or gloomy prognosis [3,4,5].

Facing death and discussions on the quality of dying have become indisputably necessary in order to offer greater understanding and the concreteness of these elements until now has been linked to individual and family experiences [2]. The need to understand such phenomena and, above all, qualify them constitutes a daunting challenge [2,6]. Thus, different methods have been adopted to measure the context of the dying process. This generally directs the eye of the researcher to family perceptions, as they tend to portray the process of death by indicating crucial elements that can qualify it as a positive experience or not, even posthumously [6,7].

Quality of Dying and Death (QODD) is an instrument with an operational and conceptual model that was first developed in 2001 by researchers of the University of Washington in the city of Seattle, USA. The scale was created to measure the quality of death and dying as well as comprehend the perceptions of family members and/or caregivers regarding the death of a loved one [8,9].

The initial instrument was composed of 31 items divided among six domains. The total score ranged from 0 to 100, with higher scores being indicative of greater quality. During the initial validation process, the domain scores had an average of 67.36 points and Cronbach’s alpha coefficient for the total score was 0.86 [8,10]. A subsequent study was conducted in 2010 with family members and friends of 205 decedents in Missoula, Montana to construct a model of latent variable domains underlying the Quality of Dying and Death (QODD) questionnaire [11].

Due to its relevance in the context in which it is applied, the QODD has been translated and validated for several languages, including Spanish, German, Chinese, and Persian, with versions containing 31, 25, and 14 items [4,5,12,13].

QODD version 3.2a was created after the elimination of nine items evaluated as unsuitable for the intensive care setting, which resulted in a 22-item instrument applicable in the intensive care unit (ICU). The same classification was maintained (with a scale of zero to ten to represent a “terrible experience” or “nearly perfect experience”, respectively). In addition to these 22 items, three others were added to improve the instrument, maintaining the reliability of the original instrument [14].

Thus, QODD version 3.2a has 25 items and six domains. Twenty-two items address aspects related to the experience of the respondent in the last days of the loved one’s life and three items address the quality of care provided by the health team and quality of the dying process [8]. 

The present study intended to give continuity to the validation process to evaluate the representativeness of the QODD 3.2a as a valid instrument for measuring the construct of what it indeed proposes to measure after cultural adaptation and content validation for the Brazilian context [15].

This methodological study was carried out in a public university, the São Paulo State University (UNESP) medical school in São Paulo, Brazil, in three stages: translation and back-translation by two native-speaking independent professionals, analysis by a committee of specialists, and pre-test phase. The final version was created by seven experts after making semantic, idiomatic, and cultural changes to 16 items. The global average for CVI was 0.99, indicating an acceptable value and adequate representation in this study (≥0.80) [15].

Thus, the aim of this study was to analyze the psychometric properties of the Brazilian Portuguese version of the QODD 3.2a for family members of patients who died in adults intensive care units.

## 2. Methods

### 2.1. Study Design

A methodological study was conducted at three public hospitals in the state of São Paulo, Brazil in the period between December 2020 and March 2022. Authorization was obtained from the authors for the translation and validation of the instrument.

### 2.2. Study Population

The inclusion criteria were family members or caregivers (formal/informal), aged 18 years or older, both sexes, having accompanied the patient in the process of death occurring between 30 and 90 days earlier, and consent to participate in the study. Individuals who reported not having the emotional strength to participate in the study and those who were not located were excluded. 

Although there is no gold standard for sample size when validating a new instrument, the recommendation is that the sample should be at least four to ten times the number of items with a minimum sample of 100 individuals, to ensure an appropriate validity analysis [16]. Our final analytical sample was comprised of 326 participants.

The invitation to participate in the study was initially performed by telephone. Following acceptance, permission was requested to send the statement of informed consent by e-mail or an electronic message application. Subsequently, one of the researchers made the second telephone contact, which had been previously scheduled. If there was agreement to participate through the signed statement of informed consent, the researcher administered the QODD 3.2a that had been translated and adapted to Brazilian Portuguese.

### 2.3. Data Collection Procedure

In this instrument questions 1 to 22 are subdivided into two parts identified as “a” and “b” with the following standard answers. For purposes of analysis, answers to “b” questions, as well as questions 23, 24, and 25 were considered, as these questions have the same answer pattern. Table 1 shows the response options for the QODD 3.2a.

The total score was obtained for the classification of the patient’s experience of dying by the sum of the items and division by the number of items effectively answered. This result was then divided by ten (total score of each item) and multiplied by 100, resulting in a range of 0 to 100 points, with higher points denoting a better quality of death.

For the evaluation of temporal stability, a retest was performed with 20 family members. For this, the random selection method was adopted and telephone contact was made 7 to 14 days after the first interview.

### 2.4. Statistical Analysis

All variables were initially described. The Kolmogorov-Smirnov test was used to determine the distribution (normal or non-normal) of continuous variables. The QODD 3.2 items were evaluated in terms of percentage and the distribution of losses using Little’s test [17], as not all “b” questions were answered due to the structuring of the questionnaire. Imputation of missing data was performed using the substitution of means method.

Reliability: Internal consistency was analyzed using Cronbach’s alpha coefficient, and temporal stability (test-retest) was analyzed using the intraclass coefficient (ICC) for perfect agreement. Values > 0.7 were considered acceptable for both [18,19].

Construct validation: A confirmatory factor analysis (CFA) was performed to evaluate the construct of items, including the goodness of fit of the models adopted, which was based on the evaluation of the root mean square error of approximation (RMSEA ≤ 0.08), comparative fit index (CFI ≥ 0.90), and the chi-squared test (χ2/df < 5) [20]. Oblimin was the rotation method adopted.

An exploratory factor analysis (EFA) was performed as the instrument’s goodness of fit to the models adopted was poor. Adequacy of the sample was evaluated using the Kaiser-Meyer-Olkin (KMO) test and viability was evaluated using Barlett’s test. Values > 0.8 were considered acceptable [21]. Horn’s parallel analysis was used to determine the number of factors/components to be extracted; factors with eigenvalues greater than 1.0 were extracted. Two criteria were considered in the EFA for the maintenance of items/factors: items with absolute factor loading higher than 0.3 in only one factor and factors to which three or more items were linked [21].

The Spearman’s coefficient was used to analyze the correlation between instrument domains, and it should be greater than 0.7 to indicate a strong correlation. Values < 0.4, 0.4 to 0.6 and >0.6 were, respectively, indicative of weak, moderate, and strong correlations [22].

The data from the participants were tabulated on electronic spreadsheets and analyses were performed in software R version 4.1.1. The significance level was set at 5% (*p <* 0.05).

This study was conducted following the guidelines of Standards for Quality Improvement Reporting Excellence (SQUIRE 2.0) [23] and COnsensus-based Standards for the selection of health Measurement INstruments (COSMIN) [24].

## 3. Results

A total of 529 deaths that occurred during the study period met the eligibility criteria. However, 203 were excluded (132 due to refusals to participate and 71 due to the impossibility of locating or contacting the family member). Thus, the sample was composed of 326 participants.

Women predominated in the sample (n = 210; 64.4%). Mean age was 45 (±13.7) years. The majority were white (n = 229; 70.2%), and had a complete high school education (n = 175; 53.7%). There was a predominance of sons/daughters (n = 177; 54.3%) and spouses (n = 48; 14.7%) who did not reside with the patient (n = 64; 19.6%). Women, white respondents, those with a high school education, and mean age of 45 (±13.0) years were also predominant in the retest (Table 2).

### 3.1. Construct Validity

The analysis of psychometric properties was performed with questions that had answers ranging from 0 to 10. Little’s test was then applied for the determination of random missing data (items not answered due to the corresponding “a” answer) (*p* = 0.77). Imputation of missing data (n = 189) was then performed with the mean of the variables.

#### 3.1.1. Confirmatory Factor Analysis (CFA)

Initially, a confirmatory factor analysis was conducted with a model including seven dimensions according to a robust error estimation. However, there was a poor goodness of fit of the adjusting indices in the final model: CFI: 0.7020, TLI: 0.766, RMSEA: 0.082, Chi-squared test: 745.653, g.l = 254, and *p* < 0.001. In addition, all possible analyses were tested using the modification indexes but that did not provide better results.

#### 3.1.2. Exploratory Factor Analysis (EFA)

**Figure 1:**
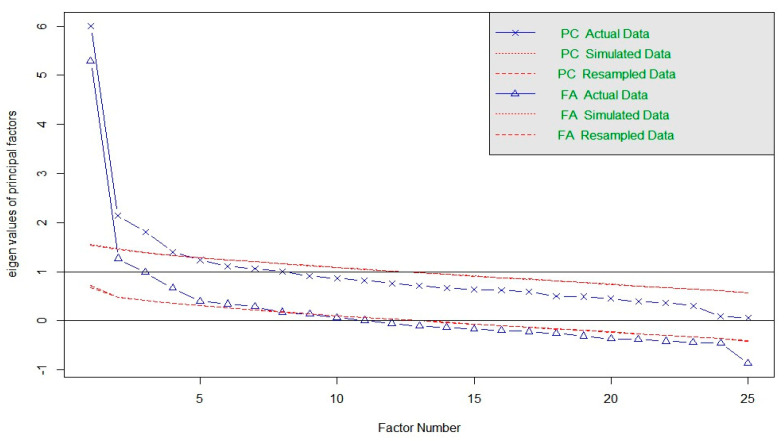
Scree plot of Horn’s parallel analysis.

The result of the KMO test was 0.87 and Barlett’s sphericity was 3090.83 (*p* < 0.0001), confirming the adequacy of the sample for EFA. Horn’s parallel analysis indicated two oblique factors with eigenvalues greater than 1.0 (Figure 1). Six outliers were detected and excluded based on the Mahalanobis distance criterion at the 0.1% level.

EFA was performed to determine how the items were grouped in relation to the factors (Table 3). However, EFA with two factors proved unviable. Upon stabilizing loadings greater than 0.3, items 1b, 2b, 7b, 8b, 19b, 24, and 25 were indicated for exclusion, as the minimum criterion established for the maintenance of a factor was having at least three variables. The sum of squares of the loadings for factor 2 was 4.705 and the proportion of variance explained was 0.2614 (chi-squared = 633.21).

“Preparation for death” was the dimension that retained the largest number of items (n = 7) in factor 2.

Table 4 displays the matrix of Spearman’s inter-item and item-total correlations, with correlations found between items and scores of the QODD 3.2a. Most of the inter-item correlations were weak to moderate. Questions 13b, 9b, and 10b were those that most presented moderate correlations with other items on the instrument: 13b and 14b/15b/16b/20b/21b; 9b and 10b/11b/15b/16b/21b; 10b and 15b/16b/23b. The other questions that had moderate correlations with two other items on the instrument were 4b and 17b; 5b and 23b; 14b and 20b; 15b and 21b; 16b and 20b; 17b and 18b/20b; 20b and 23b; 22b and 23b. When correlated with the total score, moderate correlations were found between questions 3 to 6b, 11b, 12b, 14b, 17 to 20b, and 22b. Questions 15b and 16b had a strong correlation and questions 9b, 10b, 13b, 15b, 16b, 21b, and 23b were also strongly correlated with the total score of the instrument. Regarding the AVE, only the family domain had a value higher than 0.5.

#### 3.1.3. Confirmatory Factor Analysis (CFA)

**Figure 2:**
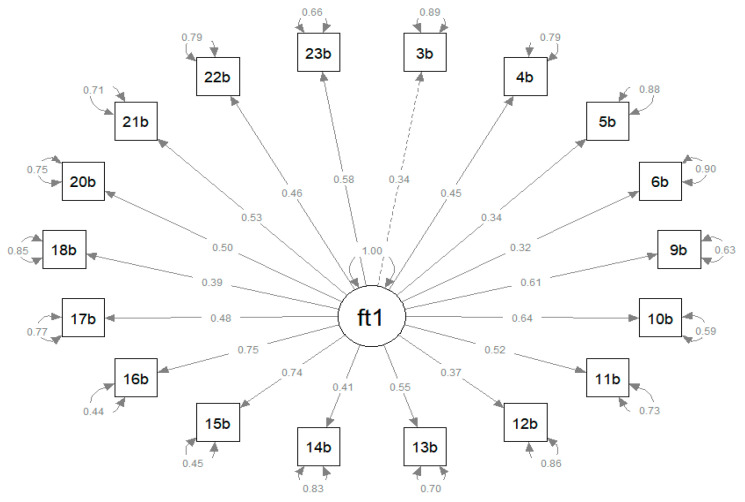
Path diagram of the confirmatory factor analysis and standardized loading of items of the version QODD 3.2a.

The analysis of the goodness of fit to the unidimensional model resulted in the following measures: CFI of 0.7545 and TLI of 0.690 (both lower than 0.9), chi-squared = 767.33, df = 135, RMSEA of 0.121 (above the limit of 0.08), with 90%CI (0.113–0.129), and *p* = 5.04409 (Figure 2).

### 3.2. Reliability 

The reliability analysis was based on the internal consistency of the instrument using Cronbach’s alpha and temporal stability (test-retest) using the ICC. Cronbach’s alpha was 0.8587 and the ICC was 0.9927 (95%CI: 0.9852–1.0002), revealing the adequate reliability of the instrument. Table 5 lists the items that composed the final 18-item version of the instrument:

## 4. Discussion

The assessment of the quality of death encompasses different conceptions and is founded on circumstances involving preparation, coping, the appreciation of identities, and responsibilities in the care provision scenario. These elements as well as sociocultural aspects tend to directly result in new meanings and experiences for those involved [3]. 

It is necessary to consider the dualities of perceptions regarding the care provided in an intensive care setting, because at the same time that the ICU deals with severe conditions, it also has a health team that provides uninterrupted care, which may represent a mitigating condition which creates a sense of security on the part of family members [4,25].

The present study had a contingent of 326 subjects. This number is higher than that reported in similar studies [4,5,12,13], in which the number of participants ranged from 72 to 150. Thus, one may infer that the Brazilian version transcended the recommendations of the literature regarding the sample equivalence of 10 individuals per item on the instrument being evaluated [16], reaching a quantity that was twofold greater than that considered necessary for the proposed validation study. However, the literature also recommends that complex instruments have a population of at least 200 subjects [12], which constitutes a good practice in the validation process of the QODD 3.2a in Brazilian Portuguese.

The number of participants in the present study was higher than that of other studies involving the scenarios of death and end-of-life care [4,5,12,13]. It is noted that it was similar to two studies developed with the Spanish [12] and Chilean populations [13], both with the same sociodemographic profile.

Similar to another German study [26], it could be seen that the assessment of the quality of death covered different conceptions and emphasized experiences of preparation, coping, symptom control, and end-of-life care. Although patients themselves are often unable to provide information about the perceived quality of their care, their closest relatives were able to assess the last days of their loved ones and contribute with undoubtedly necessary reflections, even if done posthumously.

With regards to the instrument’s adjustment model, the multivariate normality of the data and the quality measures were adequate (CFI close to 0.9 and RMSEA below 0.08), similar to the results found in the Chinese version of the QODD [5], with CFI and RMSEA of 0.93, 0.91, and 0.033.

However, it is important note that the result of a single index should not be criterion for qualifying the model as adjusted or not, rather there should be a combination of multiple indexes [27].

To obtain better parameters in terms of the quality of the construct, the validation process of the Chilean version [5] also had a second CFA based on the 13-item model suggested by Downey et al. [11]. Thus, a shorter version with only four domains was developed. Furthermore, Pearson’s correlation coefficient indicated a weak correlation when exploring the relation between each item and the factor studied, as also found in the Chinese version of the instrument [5]. 

With regards to reliability, internal consistency determined using Cronbach’s alpha was satisfactory. Similar data were found in the validation of other versions, with the Cronbach’s alpha ranging from 0.60 to 0.88 [4,5,12,13]. These findings demonstrate that the instrument is reliable for the analysis of the construct that it proposes to measure. One should bear in mind that Cronbach’s alpha coefficients are strongly influenced by the number of items on an instrument [24] and that, although the versions of the QODD have presented a factorial structure with a variable number of items, internal consistency remained satisfactory. For temporal stability, the ICC was highly significant for all items. Similar data were reported in other versions (ICC ranging from 0.88 to 0.97) [4,13].

The validation of the QODD 3.2a will enable analyses using a quantitative method in a setting that has mainly been investigated using qualitative approaches given the subjectivity of the experience of death. Besides being less costly and less complex than the development of a new instrument [18], its validation enables the exploration of primary experiences, whose constructs remain under the same standpoint of analysis, but adapted to Brazilian culture, as previously done for other languages, such as Spanish, German, Chinese, and Persian, with versions containing 31, 25, and 14 items [4,5,12,13].

As the translation and cultural adaptation of the QODD 3.2a [15] has consisted only of the first step of the process to make it reliable and suitable for application to the Brazilian context, the present study confers continuity to the psychometric validation process to certify its representativeness as a valid method for measuring the construct that it indeed proposes to measure. The entire validation process of an instrument in a different cultural context requires a rigorous psychometric validation process, including the definition of levels of standardization for evaluation, but must excel through evidence ensuring that the instrument is useful as a measurement tool.

Well-planned end-of-life care that considers the expectations in the decisions related to the patient’s treatment contributes considerably to improvements in the psychological preparedness of someone facing death. This process should respect the existing affective ties of the family, spiritual leader, and friends. They are important people who exert a positive effect on the experience of everybody involved in this delicate moment [4,5].

Faced with the magnitude of the terminality of life and the possibility that some patients may evolve into therapeutically uncontrollable stages, it is essential to adopt measures in favor of quality at the end of life, in a planned and humane way [2,28]. To this end, it is essential to monitor the entire evolution of the terminality process and establish lines of care that avoid obsolete extensions, prioritizing symptom control, and maintaining a welcoming environment to strengthen family ties [28].

### Limitations

Among the limitations of this study, we highlight the investigation difficulties imposed by the COVID-19 pandemic such as remote data collection and family members who had many restrictions on visits to the intensive care units. Another potential limitation is due to the fact that the instrument does not present a single metric of item evaluation and the possibility of lack of response to “b” items when a certain experience has not been perceived, which may have weakened the analysis.

## 5. Conclusions

The results of the present study indicate that the Brazilian Portuguese version of the Quality of Dying and Death—version 3.2a (intensive therapy) has a unidimensional structure and acceptable reliability. However, it did not obtain a good fit to the proposed factorial model.

Further exploratory studies with this instrument are needed with a duplicated sample, as the scale has different standard answers for the items and the participants may not answer “b” questions.

Nonetheless, a strong point is the availability of an unprecedented instrument in Brazil, the aim of which converges with current care guidelines that address death as a fundamental element that has an impact on posthumous perceptions of end-of-life care, indicating points suitable for intervention through the specialization of care in intensive therapy. 

## Figures and Tables

**Table 1:** Response options for the QODD 3.2a.

Questions 1–10a	Answers are scored on a scale from 0 to 6
Questions 11–21a	Answers are scored on a scale ranging from 0 (yes) to 3 (I don’t know)
Question 22a	the participant indicates how their loved one was at the moment of death using a nominal scale ranging from 1 (awake) to 4 (I don’t know)
Questions 1b–22b	Answers are scored on a Likert scale ranging from 0 (terrible experience) to 10 (nearly perfect experience) but may not be answered depending on the answer given in part “a” of the item
Questions 23, 24 and 25	Do not have subdivisions and the answers also range from 0 to 10

**Table 2:** Sociodemographic characteristics of participants in different phases of study.

Variables	Validation (n = 326)	Retest (n = 20)
Age *	45 (±13.7)	45.5 (±13.0)
Sex **		
Male	116 (35.6%)	5 (25%)
Female	210 (64.4%)	15 (75%)
Sex of loved one **		
Male	190 (58.3%)	8 (40%)
Female	136 (41.7%)	12 (60%)
Brazilian nationality **		
Yes	324 (99.4%)	19 (95%)
No	2 (0.6%)	1 (5%)
Ethnicity **		
White	229 (70.2%)	12 (60%)
Black	23 (7.1%)	1 (5%)
Asian	2 (0.6%)	0 (0.0)
Brown	69 (21.2%)	7 (35%)
Indigenous	2 (0.6%)	0 (0.0)
Other	1 (0.3%)	0 (0.0)
Schooling **		
Illiterate	27 (8.3%)	4 (20%)
Primary school	34 (10.4%)	2 (10%)
High school or trade school	175 (53.7%)	12 (60%)
Higher education	72 (22.1%)	1 (5%)
Postgraduate degree	18 (5.5%)	1 (5%)
Degree of relatedness **		
Spouse or companion	48 (14.7%)	2 (10%)
Son/daughter	177 (54.3%)	8 (40%)
Brother	22 (6.7%)	1 (5%)
Parent	8 (2.5%)	9 (45%)
Other relative	67 (20.5%)	0 (0.0)
Friend	4 (1.2%)	0 (0.0)
Resided with loved one **		
Yes	48 (14.7%)	15 (75%)
No	64 (19.6%)	5 (25%)
Knew loved one for (years) *	37.7 (±16.2)	34.4 (±15.0)

* Mean (SD)/** n (%).

**Table 3:** Factor loadings, communality, and percentage of variance of items in relation to factors.

Item	Factor 1	Factor 2	Communality	Percentage of Variance
1b		0.22	0.048	0.95
2b		0.28	0.078	0.92
3b		0.39	0.150	0.85
4b		0.47	0.217	0.78
5b		0.38	0.141	0.86
6b		0.33	0.108	0.89
7b		0.28	0.076	0.92
8b		0.16	0.025	0.97
9b		0.62	0.380	0.62
10b		0.64	0.407	0.59
11b		0.52	0.272	0.73
12b		0.34	0.118	0.88
13b		0.57	0.325	0.68
14b		0.39	0.153	0.85
15b		0.69	0.477	0.52
16b		0.70	0.488	0.51
17b		0.47	0.224	0.78
18b		0.38	0.142	0.86
19b		0.30	0.090	0.91
20b		0.50	0.252	0.75
21b		0.55	0.303	0.70
22b		0.44	0.191	0.81
23		0.60	0.364	0.64
24	0.27		0.963	0.037
25	0.29		0.919	0.081

**Table 4:** Spearman’s inter-item and item-total correlations.

Items		3b	4b	5b	6b	9b	10b	11b	12b	13b	14b	15b	16b	17b	18b	20b	21b	22b	23b
3b	rho	1.00	0.36	0.24	0.10	0.29	0.22	0.18	0.08	0.32	0.15	0.18	0.20	0.30	0.20	0.25	0.33	0.24	0.23
3b	p	0.00	0.00	0.02	1.00	0.00	0.04	0.26	1.00	0.00	1.00	0.74	0.35	0.00	0.28	0.01	0.00	0.01	0.01
4b	rho		1.00	0.37	0.11	0.38	0.38	0.29	0.15	0.30	0.21	0.35	0.32	0.46	0.14	0.31	0.34	0.29	0.30
4b	p		0.00	0.00	1.00	0.00	0.00	0.00	0.79	0.00	0.27	0.00	0.00	0.00	1.00	0.00	0.00	0.00	0.00
5b	rho			1.00	0.35	0.31	0.31	0.24	0.09	0.17	0.25	0.17	0.18	0.07	0.21	0.28	0.27	0.11	0.42
5b	p			0.00	0.00	0.00	0.00	0.04	1.00	0.27	0.30	0.76	1.00	1.00	0.42	0.01	0.01	1.00	0.00
6b	rho				1.00	0.23	0.37	0.25	0.12	0.21	0.20	0.23	0.22	0.18	0.30	0.19	0.28	0.12	0.22
6b	p				0.00	0.02	0.00	0.02	1.00	0.03	0.47	0.10	0.15	0.67	0.00	0.91	0.01	1.00	0.30
9b	rho					1.00	0.60	0.53	0.16	0.40	0.33	0.52	0.52	0.23	0.15	0.27	0.45	0.26	0.35
9b	p					0.00	0.00	0.00	0.41	0.00	0.00	0.00	0.00	0.01	1.00	0.00	0.00	0.01	0.00
10b	rho						1.00	0.35	0.37	0.39	0.34	0.50	0.53	0.36	0.33	0.39	0.36	0.25	0.41
10b	p						0.00	0.00	0.00	0.00	0.00	0.00	0.00	0.00	0.00	0.00	0.00	0.02	0.00
11b	rho							1.00	0.11	0.34	0.14	0.37	0.38	0.20	0.11	0.18	0.33	0.28	0.28
11b	p							0.00	1.00	0.00	1.00	0.00	0.00	0.05	1.00	0.27	0.00	0.00	0.00
12b	rho								1.00	0.16	0.24	0.34	0.32	0.23	0.25	0.13	0.10	0.22	0.30
12b	p								0.00	0.30	0.02	0.00	0.00	0.01	0.03	1.00	1.00	0.02	0.00
13b	rho									1.00	0.50	0.45	0.48	0.35	0.23	0.41	0.41	0.36	0.38
13b	p									0.00	0.00	0.00	0.00	0.00	0.01	0.00	0.00	0.00	0.00
14b	rho										1.00	0.34	0.34	0.22	0.24	0.42	0.29	0.23	0.26
14b	p										0.00	0.00	0.00	0.27	0.03	0.00	0.00	0.08	0.00
15b	rho											1.00	0.93	0.33	0.25	0.37	0.40	0.34	0.38
15b	p											0.00	0.00	0.00	0.01	0.00	0.00	0.00	0.00
16b	rho												1.00	0.36	0.27	0.41	0.39	0.33	0.39
16b	p												0.00	0.00	0.01	0.00	0.00	0.00	0.00
17b	rho													1.00	0.41	0.41	0.33	0.39	0.37
17b	p													0.00	0.00	0.00	0.00	0.00	0.00
18b	rho														1.00	0.32	0.33	0.25	0.31
18b	p														0.00	0.00	0.00	0.00	0.00
20b	rho															1.00	0.39	0.38	0.49
20b	p															0.00	0.00	0.00	0.00
21b	rho																1.00	0.37	0.40
21b	p																0.00	0.00	0.00
22b	rho																	1.00	0.41
22b	p																	0.00	0.00
23	rho																		1.00
23b	p																		0.00
Score	rho																		

**Table 5:** Questions on final version of instrument validated for Brazilian Portuguese.

Item	Questions
1	How often was your loved one able to feed her/himself?
2	How often did your loved one appear to breathe comfortably?
3	How often did your loved one appear to feel at peace during the process of dying?
4	How often did your loved one appear to be unafraid of dying?
5	How often did your loved one spend time with his/her family or friends?
6	How often did your loved one spend time alone?
7	Was your loved one touched or hugged by his/her loved ones?
8	Were all of your loved one’s health care costs taken care of?
9	Did your loved one say goodbye to loved ones?
10	Did your loved one clear up any bad feelings with others?
11	Did your loved one have one or more visits from a religious or spiritual advisor?
12	Did your loved one have a spiritual service or ceremony before his/her death?
13	Did your loved one receive a mechanical ventilator (respirator) to breathe for him/her?
14	Did your loved one receive dialysis for his/her kidneys?
15	Did your loved one discuss his or her wishes for end-of-life care with his/her doctor -- for example, resuscitation or intensive care?
16	Was any family member present at the moment of your loved one’s death?
17	In the moment before your loved one’s death, was he/she:
18	Overall, how would you rate the quality of your loved one’s dying?

## Data Availability

The datasets generated and/or analyzed during the current study are not publicly available to preserve anonymity of the respondents but are available from the corresponding author on reasonable request.
